# An Unusual Presentation of Idiopathic Lymphatic Cyst of the Thigh

**DOI:** 10.1155/2018/1930207

**Published:** 2018-12-10

**Authors:** Chairat Burusapat, Sophilak Sringkarawat, Sineenard Thanapurirat, Rapeepat Sapruangthong, Chatchai Pruksapong, Kantang Satayasoontorn

**Affiliations:** ^1^Division of Plastic and Reconstructive Surgery, Department of Surgery, Phramongkutklao Hospital and Phramongkutklao College of Medicine, Bangkok, Thailand; ^2^Division of Plastic and Reconstructive Surgery, Department of Surgery, Phramongkutklao Hospital, Bangkok, Thailand; ^3^Department of Pathology and Laboratory Medicine, Phramongkutklao Hospital, Bangkok 10400, Thailand

## Abstract

Lymphatic cyst is a collection of lymphatic fluid with an epithelial lining which can either represent lymphatic malformations or may occur after trauma or operation. Idiopathic lymphatic cyst of the thigh in adulthood is extremely rare. We report a case of a 27-year-old Thai female presented with a rapidly growing mass over her right thigh. The magnetic resonance imaging scan demonstrated a well-defined, thin rim, enhancing simple cyst. The cyst disappeared for a few days after aspiration. Completed surgical resection was performed for definitive diagnosis and treatment. Immunohistochemical studies revealed the cell lining was positive for CD34, CD31, and D2-40 and negative for calretinin. To our best knowledge, idiopathic lymphatic cyst of the thigh in young adult has never been reported and should be kept in mind in the patient who presents with abnormal thigh mass.

## 1. Introduction

Lymphatic cyst is a collection of lymphatic fluid with an epithelial lining which can either represent lymphatic malformations or may occur after trauma or operation [1]. Idiopathic lymphatic cyst of the thigh, without a history of prior trauma or operation, in adulthood is extremely rare [2].

We present an unusual case of a young adult female who presented with symptomatic mass at the thigh which has not previously been reported in English literature as the origin of a lymphatic cyst.

## 2. Case Report

**Figure 1:**
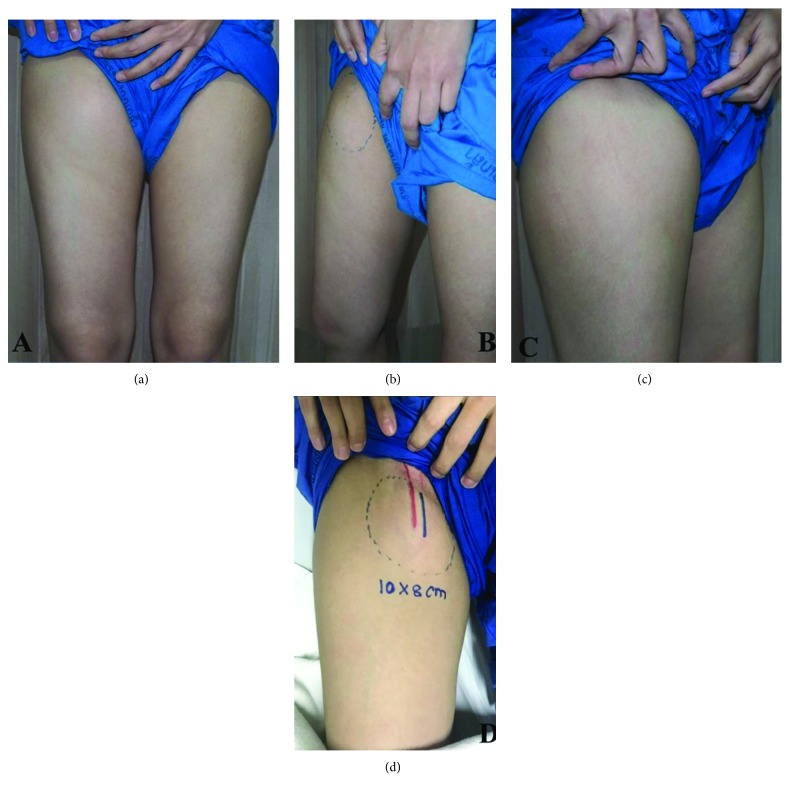
Demonstrated cystic mass at the right thigh: (a) front view, (b) medial view, (c) lateral view, and (d) outline of the mass overlying the femoral vessels.

A 27-year-old Thai female presented with a rapidly growing mass over her right thigh for 3 months. She had no underlying or previous surgery. She had pain when she walked. She had slightly limited her right hip function on flexion and abduction. Physical examination revealed a firm nonpulsatile mass over the right thigh measuring ~8 × 6 cm. in diameter (Figure 1). No notable grossly skin involvement and the mass appears not fixed to bony structure. No sensory deficit was identified. Inguinal lymph node cannot be palpated.

**Figure 2:**
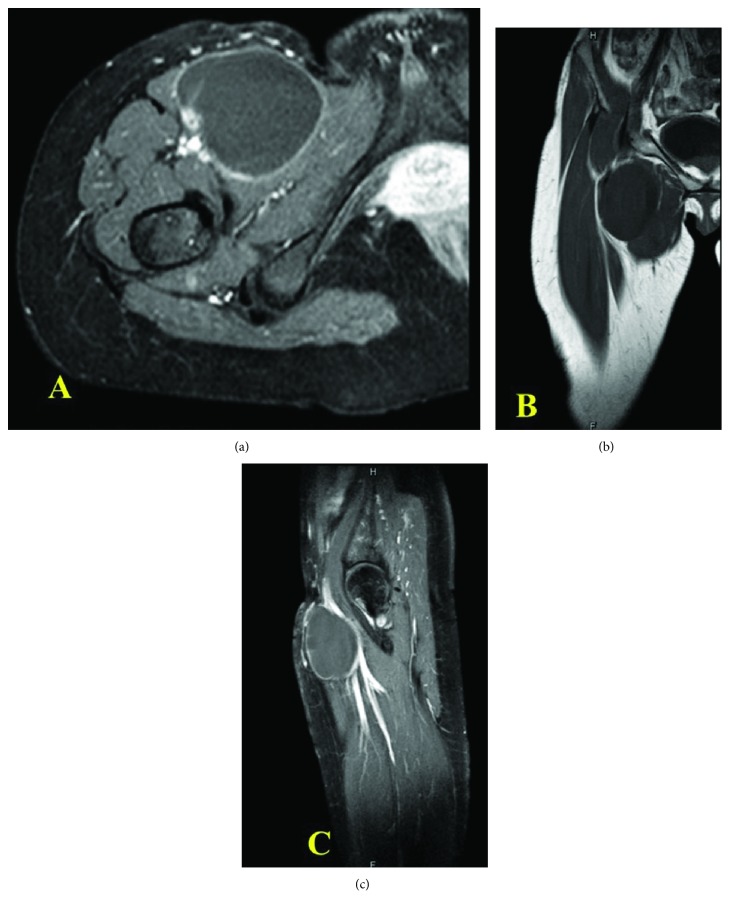
MRI shown cystic lesion: (a) axial view, (b) coronal view, and (c) sagittal view.

The magnetic resonance imaging (MRI) scan demonstrated a 7.8 × 5.8 × 5.7 cm, in vertical × transverse × AP diameter of well-defined, thin rim, enhancing simple cyst with vascularity at the right anterior intermuscular fascia of the upper thigh, just inferior to the right inguinal region. The cyst was located between the pectineus muscle and sartorius muscle, accompanied with anterior bulging to subcutaneous fat. The right common femoral artery and vein and superficial artery and vein were displaced posteriorly (Figure 2).

Percutaneous aspiration of cyst revealed clear yellowish fluid about 60 ml., and the cytological examination showed hypocellularity consisting of few small lymphocytes and foamy macrophages in background of few erythrocytes and concluded cystic fluid. The cyst disappeared for a few days and presented in the same size within one week.

**Figure 3:**
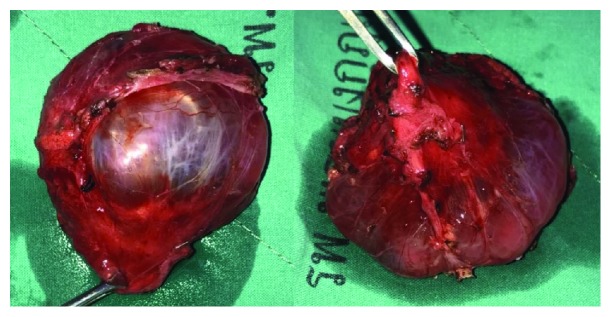
Complete surgical removal of cyst.

**Figure 4:**
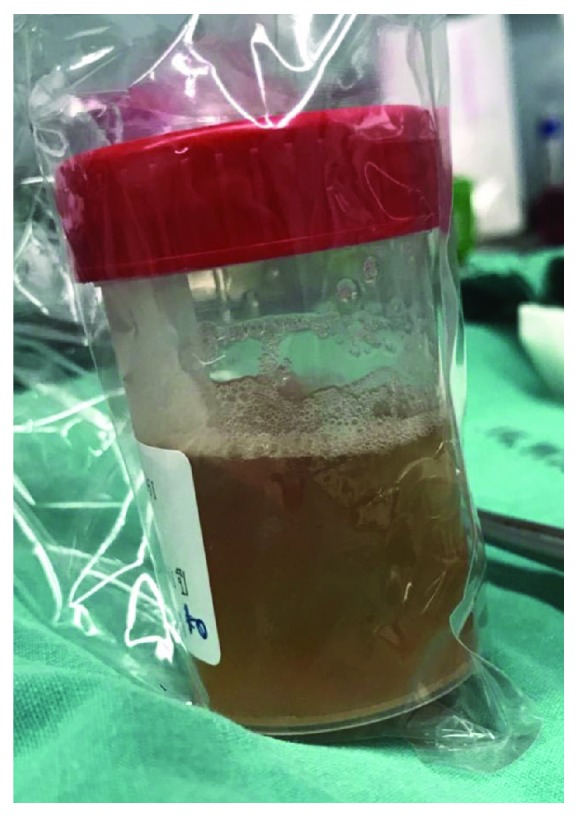
Cystic content showed the clear, yellow fluid.

The operation was performed on supine position under general anesthesia. Vertical incision was performed; the cyst was identified and found dense adherance to femoral artery. Carefully, dissection was done. Complete cystic removal with femoral artery preservation was successful with minimal leakage of cyst wall (Figure 3). Cystic content had shown the clear, yellow fluid (Figure 4).

**Figure 5:**
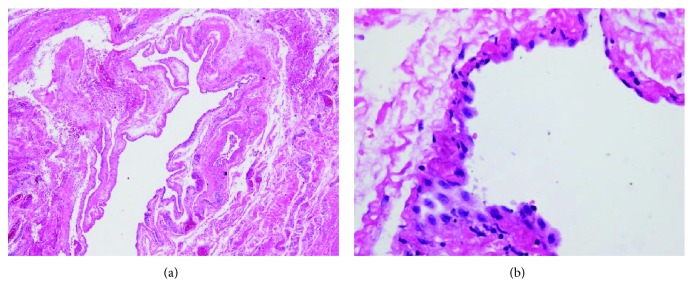
(a, b) H&E staining showed that the cystic wall is lined by a single layer of flat lining cells.

**Figure 6:**
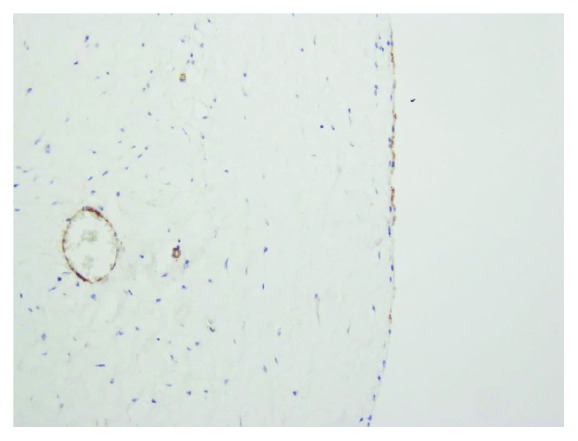
The cystic lining cells showed immunoreactive with CD31.

**Figure 7:**
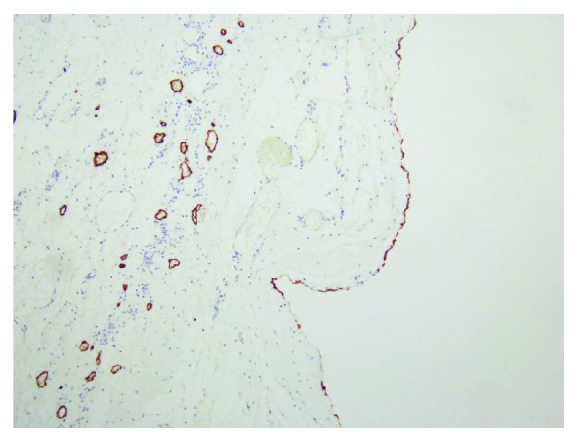
Immunohistochemical study revealed that the flattened cystic lining cells are positive for D2-40.

Microscopic examination was demonstrated as Figure 5. Hematoxylin-eosin staining (H&E staining) indicated that the wall of the cyst consisted of a single flattened cell lining. The cell lining was histologically supposed to be derived from endothelium or mesothelium by routine H&E staining (Figures 5(a) and 5(b)). Immunohistochemical studies for D2-40, CD31, CD34, and calretinin were performed to define the nature/origin of the cystic cell lining. Immunohistochemical studies revealed the cell lining of the cyst was positive for CD34, CD31 (Figure 6), and D2-40 (Figure 7) and negative for calretinin. The constellation of histology and immunohistochemistry demonstrated that the wall of the cyst was not derived from the mesothelium but from the lymphatic vessels. The final pathological diagnosis was a benign lymphatic endothelial-lining cyst of soft tissue. The cystic fluid was sent for cytology and revealed no malignancy. No postoperative complication was found.

## 3. Discussion

Spontaneous thigh mass in adolescent or young adult is extremely rare. The accurate preoperative diagnosis is difficult. The differential diagnosis of thigh mass includes lipoma, liposarcoma, aneurysm, and inguinal hernia. Although, the cyst is unilocular and filled with clear fluid, the malignant cyst cannot be excluded. The cytological examination cannot give the detail and exclude the malignancy. The absolute guideline for lymphatic cyst is not established. Total removal of the cyst is the definitive diagnosis and treatment.

Clinically, primary cysts occur predominantly in children and young adolescents; often asymptomatic until large sizes, they may then present with local or referred pain. However, rapidly swelling of thigh cyst without history of small cyst, trauma, or surgery of the thigh is rare.

Lymphatic cyst is rarely seen than lymphocele and lymphangioma; it is different from a lymphocele that is a collection of lymphatic fluid without an epithelial lining that develops in anatomical compartments, often after surgery or trauma [3].

Lymphangioma is a congenital lymphatic malformation that usually presents as a congenital mass in infancy and classified into microcystic (<2 cm) and macrocystic (>2 cm) [4]. These masses are most common in the cervicofacial region and rarely occur elsewhere in the body.

An acquired lymphangioma (lymphangiectasia) can occur after surgery or radiotherapy because of damage to the draining lymphatic channels [2].

Tagge and Baron reported the etiology of lymphatic cysts may be attributed to ectasia of the lymphatic vessels or to cystic degeneration of a hamartoma [5].

Large lymphatic cysts have been reported in adrenal gland [6]. However, idiopathic lymphatic cyst of the thigh is extremely rare. Patients with lymphatic cysts may be present with a wide range of symptoms.

The definitive diagnosis of lymphatic cyst is confirmed by immunohistochemistry. Percutaneous aspiration of the cyst under ultrasound guidance is the initial treatment modality used. However, recurrence rates of up to 80% have been reported, with repeated aspiration being a risk factor for the development of infection [7]. Instillation of sclerosants, using agents such as OK-432, bleomycin, doxycycline, acetic acid, and alcohol, has also been used in lymphatic malformation [8, 9]. However, the success of instillation of sclerosants in lymphatic cyst has not been reported.

Surgical excision is the mainstay and definitive therapeutic option and can achieve total excision and prevention of recurrence, as the cyst wall is removed.

Lymphatic cyst is considered benign with excellent prognosis [10]. Malignant transformation has not been demonstrated.

To the best of our knowledge, idiopathic lymphatic cyst of the thigh in young adult has never been reported and should be kept in mind in the patient who presents with abnormal thigh mass.

## 4. Conclusion

Lymphatic cyst of the thigh remains a rare condition and should be among the top in differential diagnosis of unilocular cyst in adolescent or young adult. Completed surgical resection, keeping the cyst intact, is recommended for definitive diagnosis and treatment. Long-term follow-up to check for recurrence and/or neoplastic transformation is recommended.
